# Optimisation of ultrasound liver perfusion through a digital reference object and analysis tool

**DOI:** 10.1186/s41747-019-0086-5

**Published:** 2019-04-03

**Authors:** Ángel Alberich-Bayarri, Jose Tomás-Cucarella, Alfredo Torregrosa-Lloret, Javier Sáiz Rodriguez, Luis Martí-Bonmatí

**Affiliations:** 1 Biomedical Imaging Research Group (GIBI2^30), Hospital Universitari i Politècnic La Fe, Avda. Fernando Abril Martorell 106, Torre A, 46026 Valencia, Spain; 2 Quantitative Imaging Biomarkers in Medicine, QUIBIM SL, Valencia, Spain; 3 Department of Electronics Engineering, Polytechnics University of Valencia, Valencia, Spain

**Keywords:** Biomarkers, Liver, Perfusion imaging, Phantoms (imaging), Ultrasonography

## Abstract

**Background:**

Conventional ultrasound (US) provides important qualitative information, although there is a need to evaluate the influence of the input parameters on the output signal and standardise the acquisition for an adequate quantitative perfusion assessment. The present study analyses how the variation in the input parameters influences the measurement of the perfusion parameters.

**Methods:**

A software tool with simulator of the conventional US signal was created, and the influence of the different input variables on the derived biomarkers was analysed by varying the image acquisition configuration. The input parameters considered were the dynamic range, gain, and frequency of the transducer. Their influence on mean transit time (MTT), the area under the curve (AUC), maximum intensity (MI), and time to peak (TTP) parameters as outputs of the quantitative perfusion analysis was evaluated. A group of 13 patients with hepatocarcinoma was analysed with both a commercial tool and an in-house developed software.

**Results:**

The optimal calculated inputs which minimise errors while preserving images’ readability consisted of gain of 15 dB, dynamic range of 60 dB, and frequency of 1.5 MHz. The comparison between the in-house developed software and the commercial software provided different values for MTT and AUC, while MI and TTP were highly similar.

**Conclusion:**

Input parameter selection introduces variability and errors in US perfusion parameter estimation. Our results may add relevant insight into the current knowledge of conventional US perfusion and its use in lesions characterisation, playing in favour of optimised standardised parameter configuration to minimise variability.

## Key points

- Input parameters of the ultrasound devices impact on the final results of quantitative perfusion analysis using microbubble-based contrast agents.
- Virtual phantoms modelling ultrasound perfusion acquisitions allow to assess the influence of the input parameters in perfusion measurements by the creation of digital reference objects with known output values.
- The lowest correlation when different software were used was obtained for mean transit time and area under the curve parameters due to different washout analysis, while maximum intensity and time to peak were similar and showed a high correlation with ground truth.

## Background

Ultrasonography (US) is a well-established and highly accessible medical imaging modality. It has significantly evolved over time, incorporating the use of contrast agents to improve tissue and lesion contrast as well as allowing dynamic perfusion studies. US contrast media have opened a new panorama for quantitative imaging, significantly expanding US clinical applications in several organs such as liver, breast, and heart diseases, among many others [1–4].

In these perfusion studies, a contrast agent based on microbubbles is injected intravenously and image is captured in real time [5–7]. Like in other medical imaging modalities, such as magnetic resonance imaging and computed tomography, time-intensity curves (TICs) can be extracted from the voxel signal variation. Nevertheless, due to microbubble size, they do not reach interstitial space and all models consist of a uni-compartment approach. A key aspect to the TIC-based quantification is the assumption that the variation in intensity is proportional to the concentration of the contrast agent and therefore related to blood flowing properties [8]. Nevertheless, signal is also influenced by technical parameters related to the image acquisition procedure, such as gain, dynamic range, or transducer frequency, and by patient-related characteristics.

Regarding the technical parameters, currently, the US quantification of tissue perfusion has important challenges. Nowadays, efforts are being made for the standardisation of US devices for dynamic contrast-enhanced examinations by using calibration methods and guidelines [9–11]. Factors such as the system configuration, the position and orientation of the probe, and the injection rate of the microbubble-based contrast agent influence the quantitative analysis. There are also significant variations in the TIC results as a consequence of the loss of spatial coherence due to transducer repositioning during the dynamic examination [12]. Even during the same acquisition, areas within the same organ, and therefore with similar perfusion profile, may generate different TICs [8, 9, 13–19]. Those technical factors that may contribute to inaccuracies and errors in US quantitative measures can be related to the US equipment settings and aspects related to the type and preparation of the microbubble-based contrast agent [20]. Patient-related factors include different physiological conditions of the subject which imply different propagation/attenuation factors and also the breathing and breath-hold collaboration. Both technical and patient-related factors limit the consideration of US perfusion parameters as quantitative imaging biomarkers.

The aim of our work was to analyse the relationship between the system input parameters and the variability in the perfusion measurements in order to optimise the acquisition of images and US perfusion analysis.

## Methods

**Fig. 1:**
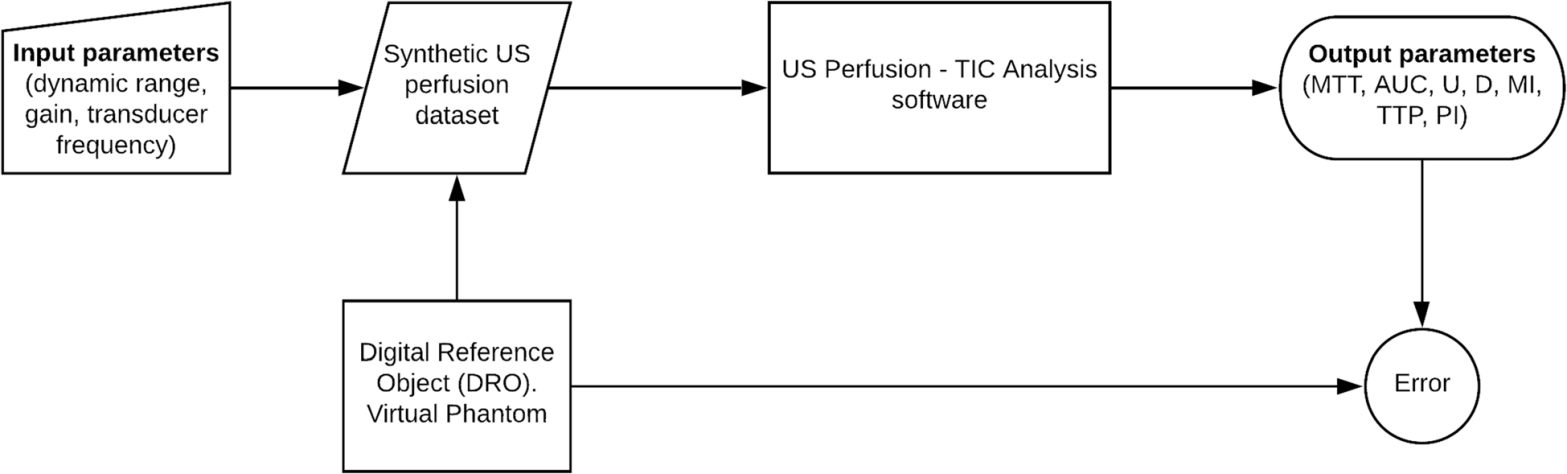
Schematic diagram of the process for theoretically obtaining the optimum input parameters minimising output variability. *AUC* area under the curve, *D* downslope, *MI* maximum intensity, *MTT* mean transit time, *PI* perfusion index, *TIC* time-intensity curve, *TTP* time to peak, *U* upslope, *US* ultrasound

In order to simulate the US signal and the behaviour of the contrast agent and evaluate the influence that different parameters may have in the measurements, an US perfusion simulator (virtual phantom) and a software tool for perfusion quantitative analysis were engineered and developed. All the algorithms and software tools were developed using Matlab r2013a (Mathworks Inc., Natick, MA, USA). The software was designed to be applied to any US perfusion dataset with independence of the manufacturer of the system (Fig. 1). All the analysis was performed using a workstation computer (Intel Xeon 64 bits, 3.3 GHz, 4 Gb of memory).

The developed US perfusion simulator was applied to generate different datasets with varying acquisition characteristics and evaluate the optimum input parameters minimising the variability of the perfusion parameters.

After the optimum input parameters were calculated, these were introduced in a real US system and the perfusion results obtained in a group of patients were compared to the values provided by an already existing commercial solution (Contrast Dynamics Software®, Siemens AG, Erlangen, Germany), which was considered as the reference pattern, despite the details of the proprietary calculation algorithms applied to the images were unknown.

### Development of US perfusion virtual phantom

A US perfusion virtual phantom was synthesised by simulating the US signal in order to analyse the influence of the acquisition parameters in the measurement of the mean transit time (MTT), the area under the curve (AUC), the maximum intensity (MI), and the time to peak (TTP) under stable conditions.

**Fig. 2:**
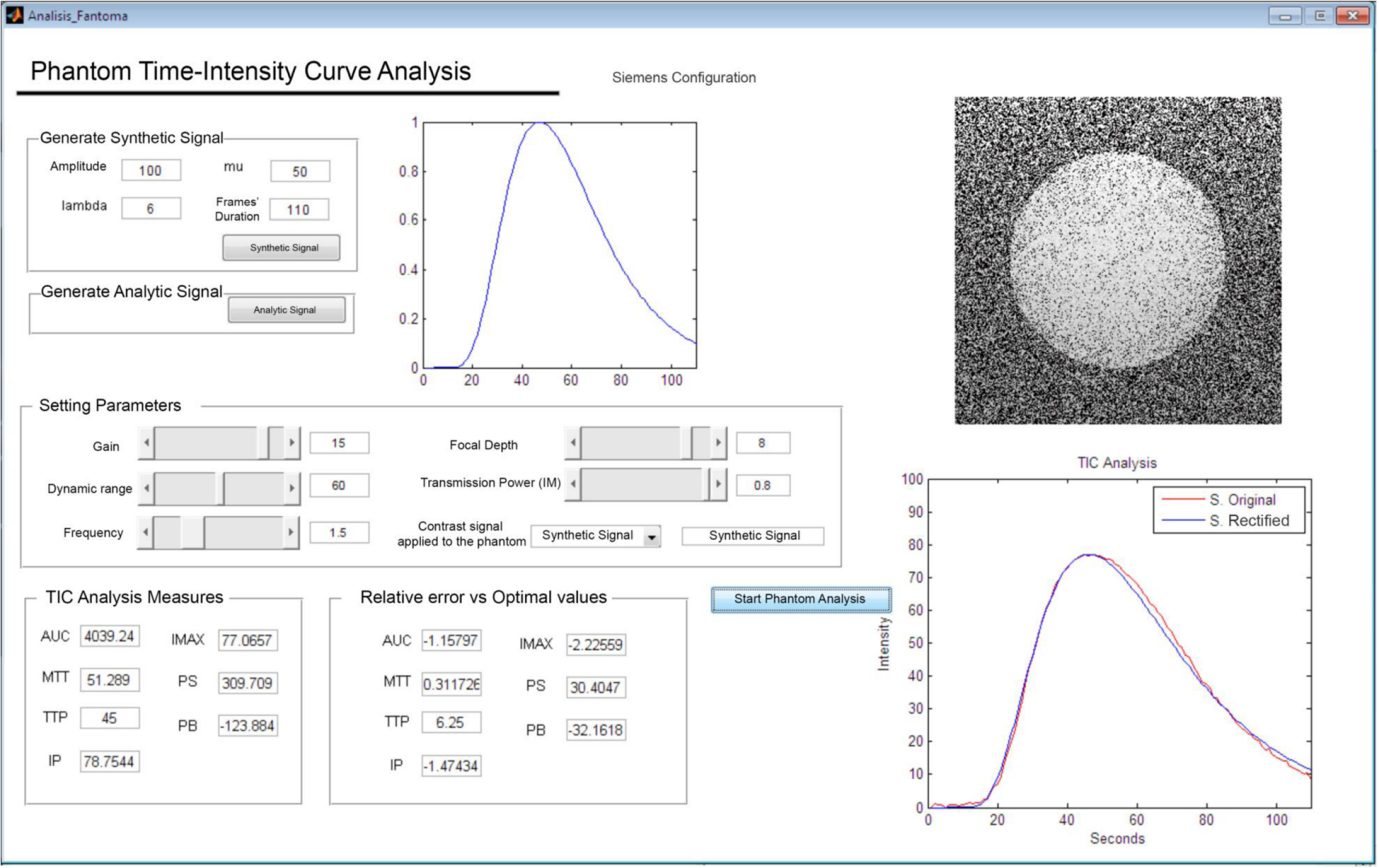
User interface of the US perfusion digital reference object and signal simulator

The ranges of the available input parameters in real US acquisition devices were specified for the US simulator [21]. Sequences of images were synthesised in the simulator in order to observe how input parameters influence MTT, AUC, MI, and TTP calculations by changing gain, dynamic range, focus, frequency, and mechanical index settings (Fig. 2).

The phantom synthesis methodology creates a series of images simulating the contrast enhancement through time, produced by the microbubble-based contrast circulation through the vessels. The basic unenhanced sequence was designed to have a length of 200 frames and a size of 512 × 512 pixels per frame. A Gaussian noise with a 0 mean and a variance of 0.3 was considered, as frequently used in computer vision experiments [11]. The 0 mean allows to not introduce an offset in the main signal values and the variance of 0.3 to introduce a significant amount of noise in the images. To simulate enhancement, a parabolic velocity profile of the microbubble-based contrast agent was contemplated. In order to model the US signal, the pressure mathematical expression shown in Eq. 1 was used.

1 $$ v(t)=\left|k\int R(z)a\left(t-\frac{2z}{c}\right){e}^{-2z\propto \left({f}_o\right)}{e}^{i{w}_o\left(t-\frac{2z}{c}\right)} dz\right| $$

where the voltage at time *t* was related to the sum of all signals received at that temporal instant, *z* is the depth achieved by the signal, *R*(*z*) are reflection regions crossed by the signal, *α* represents the attenuation of the signal because of the medium, and *f*_*o*_ is the transmitting frequency.

After demodulation of the received echoes, the transducer converts pressure measurements into voltage. Subsequently, the signal is quantified and represented as a greyscale. Spatial registration is required to analyse the simulated data-compressed images of 8-bit. The data-compressed images are applied a logarithmic compression (see Eq. 2), followed by a linearisation (see Eq. 3).

2 $$ \mathrm{QL}(V)=\mathrm{uint}8\left[\left({2}^8-1\right)\frac{20\mathrm{dB}}{{\mathrm{LC}}_{\mathrm{DR}}}{\log}_{10}\left(\frac{V}{V_{\mathrm{max}}}{10}^{\frac{{\mathrm{LC}}_{\mathrm{DR}}}{10\ \mathrm{dB}}}\right)\right] $$

3 $$ \mathrm{EP}\left(\mathrm{QL}\right)={\left({V}_{\mathrm{max}}{10}^{\left(\frac{\mathrm{QL}}{2^8-1}-1\right)\frac{{\mathrm{LC}}_{\mathrm{DR}}}{20\mathrm{dB}}}\right)}^2 $$

These conversion procedures were also introduced into the simulator.

In these expressions, *V*_max_ is the maximum echo amplitude of the raw radiofrequency data which was set at 2^15^ – 1 (maximum positive amplitude of signed 16-bit integer), *V* is the echo-amplitude, LC_DR_ is the dynamic range of log-compression expressed in dB, and uint8 represents the unsigned 8-bit integer quantification typecast operator.

For the normal procedure of the simulator, the compression and linearisation logarithm was applied to the image sequence received from the virtual phantom.

In order to simulate the input curves and the volume dose of contrast agent, the local density random walk (LDRW) model [20] was applied. The LDRW model represents the concentration of contrast over time, as it can be observed in Eq. 4.

4 $$ C(t)=\frac{m}{Q}{e}^{\lambda}\sqrt{\frac{\lambda }{2\pi \upmu t}{e}^{\frac{-\lambda }{2}\left(\frac{t}{\mu }+\frac{\mu }{t}\right)}} $$

where *m* is the injected mass of the contrast agent, *Q* is the volumetric flow, *λ* is a parameter related to the diffusion constant of the system, and *μ* is the average time of flight of the contrast to arrive from the injection entry to the detection site.

### US perfusion analysis

**Fig. 3:**
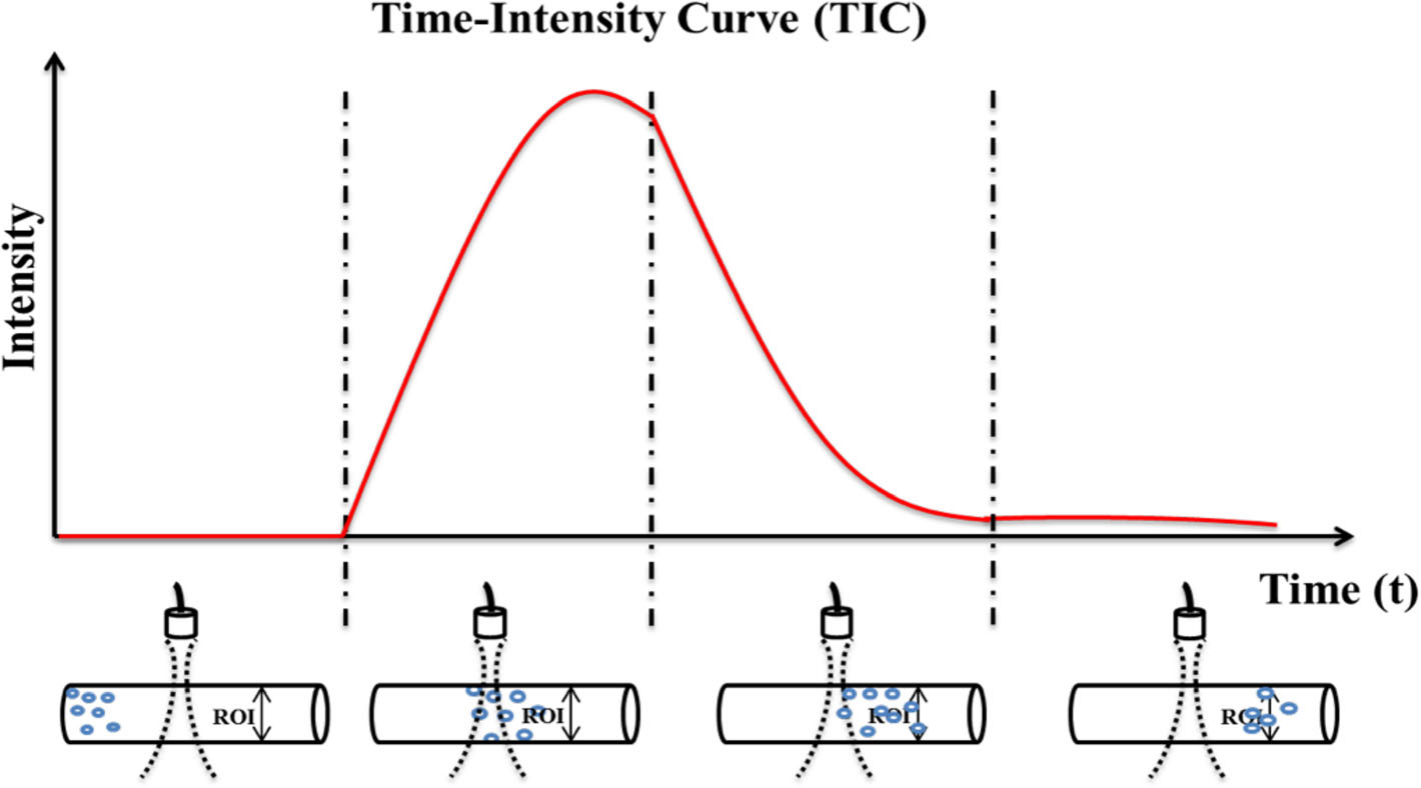
Registration of the time-intensity curve. When the contrast has not reached the field of view of the transducer, the signal intensity is at baseline. Once the contrast in the vessels reaches the region being scanned, the transducer receives reflected waves caused by microbubbles and an increase in the signal is observed. As the contrast disappears, the signal intensity diminishes

The TIC measured in liver perfusion acquisitions of the study followed a one-compartment intravascular pattern (Fig. 3). In the curve analysis algorithms, three different pharmacokinetic phases were considered. First, an arterial phase corresponded to the bolus arrival of the microbubble-based contrast agent to the arterial circulation which takes a few seconds after the intravenous injection of the contrast agent. This period lasted about 30–35 s. The second phase, named as portal or venous phase in the liver, was then initiated, lasting up to 120 s. Final plateau late phase corresponded to contrast elimination [5]. The signal from the microbubble-based contrast agent disappeared completely after 240–360 s. Algorithms for the identification of these curve phases were implemented. This allowed the calculation of MTT, AUC, MI, and TTP.

All the developed methods were implemented in a user interface whose primary function was to analyse the perfusion properties of the contrast bolus in the organism.

**Fig. 4:**
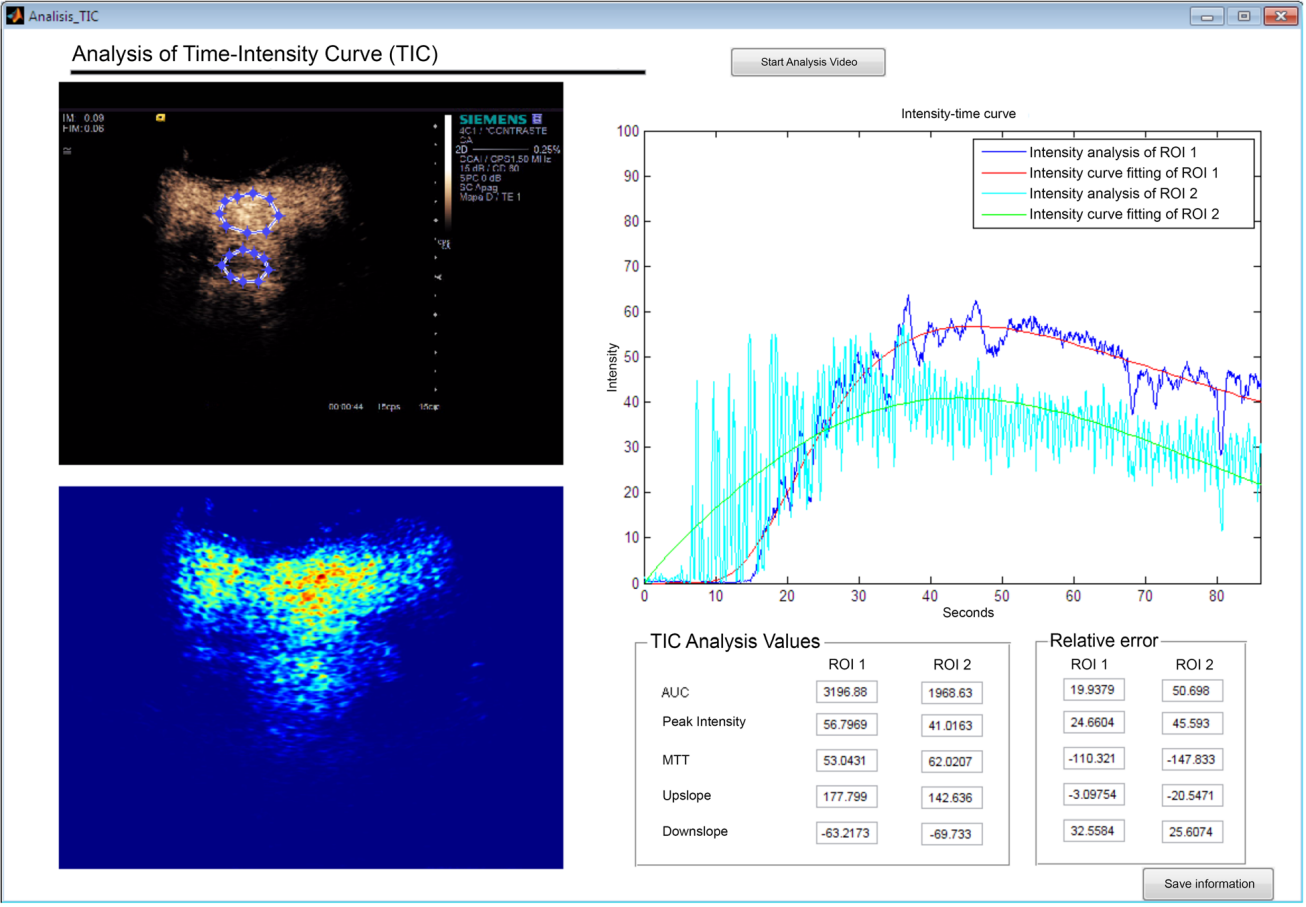
User interface of the perfusion analysis tool. The user interface contains axes to visualise the time-intensity curve (TIC) and the results of the analysis in two different ROI. It also includes the possibility of visualising the ultrasound video for regions of interest delineation

Once the study was loaded, a region was delimited in the tissue or lesion under study (Fig. 4). The curve obtained was adjusted to a Gaussian distribution by LDRW model.

The analysis tool allowed to analyse log-compressed greyscale video in “.avi” format, exported off-line from US devices.

### Optimisation of input parameters

In order to evaluate the dependency of the results on the input parameters and, therefore, to choose an optimum acquisition protocol, four different experiments were performed by varying specific parameters while maintaining the other at constant values. The details of the experiments and the steps of variation of the different parameters can be appreciated in Table 1. A total of 109 combinations of the different parameters was evaluated.

**Table 1:** Input parameters and corresponding variations introduced to the system

Experiment number	Parameter	Range of variation	Step
1	Gain	- 20 to 20 dB	1 dB
2	Dynamic range	30 to 90 dB	5 dB
3	Gain and dynamic range	- 20 to 20 dB	1 dB
		30 to 90 dB	5 dB
4	Frequency	1 to 3 MHz	0.5 MHz

The output signals of the experiments were measured, and the errors in MTT, AUC, MI, and TTP were analysed. The input parameters were optimised considering the minimum possible error among all the measurements obtained.

### *In vivo* analysis in patients

A total of 13 patients (8 males and 5 females, mean age 58 ± 7 years, paired to age) diagnosed with cirrhosis and liver hepatocarcinoma by previous magnetic resonance imaging and pathology were consecutively included in the study after receiving Ethics Committee approval for the project. Each enrolled subject signed the informed consent. Recruitment period was of 6 months. All the US acquisitions were performed using an Acuson S2000 system (Siemens, Erlangen, Germany). The process of image acquisition and injection of the contrast agent (SonoVue®, Bracco) was identical all over the study. The equipment was configured by adjusting the output power (mechanical index), frequency, gain, dynamic range, depth, zoom, and frame rate [22, 23]. Specifically, optimum gain, dynamic range, and frequency parameters derived from the US perfusion simulation study were considered. In order to minimise the influence of patient conditions and physiological motion, subjects were instructed to have a smooth breathing during the acquisition. After intravenous injection of 2.4 mL of SonoVue® (Bracco, Milan, Italy), the liver was examined for 3 min by an experienced radiologist with more than 30 years of experience with liver US. The region studied consisted of the one where the lesion was present. Finally, the video data of the US perfusion sequence was acquired and stored in “.avi” format.

Data was analysed using both the in-house developed US perfusion analysis tool and the clinically approved commercial software, which was considered as the reference. The regions of interest were visually verified to be positioned in the same location. The calculation of the optimum parameters was performed by an algorithm randomly selecting input parameter combinations; for this, a random command provided a random number that was used to select the position of the vector of starting variables.

### Statistical analysis

Data normality was verified by means of the Kolmogorov-Smirnov test. For the comparison against a ground truth consisting of a commercial software, the relative error was calculated. The relative error of the measurements obtained from the analysis comparing to the synthesised parameters in the digital reference object (considered as the reference or ground truth) was obtained by calculating the difference between measurement and ground truth and dividing it by the latter, finally multiplying by 100 to obtain a percentage. For better understanding the relationships between pairs of results using both in-house and commercial methods, Pearson correlation, linear regression, and Bland-Altman techniques were used. A *p* value lower than 0.05 was considered as the threshold for statistical significance for all tests. SPSS (version 24; SPSS, IBM, Chicago, IL) was used for the analysis.

## Results

### Optimisation of acquisition

The maximum errors obtained, considering all the range of input parameters, were 3.30% for MTT, 17.02% for AUC, 11.4% for MI, and 1.05% for TTP. For the variation in gain, frequency, and dynamic range, an almost null of approximately 0% for all the parameters was obtained at 0 dB, 1.5 MHz, and 60 dB, respectively. After parameter optimisation, it was found that the 0 dB gain, although providing the lowest error, would not provide human readable images in clinical routine by the reduced pixel intensity of the images. Therefore, the optimum gain value to both minimise error and allow clinical utility was set at 15 dB, within the range of frequently used values in clinical practice. The values of 15 dB for gain, 1.5 MHz for frequency, and 60 dB for dynamic range introduced an uncertainty of 1.48% for MTT, 1.43% for AUC, 1.16% for MI, and 1.32% for TTP.

### *In vivo* analysis methodology validation

There was a moderate variation between the measurements obtained with both software solutions. The values obtained for the MTT, AUC, MI, and TTP parameters followed a normal distribution. The relative errors obtained can be appreciated in Table 2. The average relative error between the in-house implemented methodology and the commercial solution in the calculated parameters was below 5%. When absolute values of the relative errors were considered, the average absolute difference between both analysis solutions reached 24% for the AUC parameter, while it was below 13% for all the remaining parameters.

**Table 2:** Relative errors calculated between the in-house and the commercial software for the analysed parameters, including mean relative error for every parameter and mean relative error considering the absolute values

Case number	Relative error (%)			
	MTT	AUC	MI	TTP
1	7.08	- 12.12	5.50	5.84
2	- 1.94	12.01	- 3.55	6.41
3	- 8.94	25.27	- 17.13	- 1.13
4	- 1.57	- 9.05	- 2.25	28.64
5	- 25.89	22.68	- 37.78	17.29
6	- 4.80	49.91	- 8.66	- 24.64
7	10.53	22.66	17.36	- 13.79
8	- 9.63	- 64.09	- 3.87	- 29.61
9	- 19.58	- 40.41	- 23.16	9.71
10	16.39	12.88	21.11	9.31
11	- 6.34	15.59	- 7.57	- 4.80
12	18.57	8.08	14.86	5.66
13	8.52	18.91	4.46	3.84
Mean	- 1.35	4.79	- 3.13	0.97
Mean absolute	10.75	24.13	12.87	12.36

*MTT* mean transit time, *AUC* area under the curve, *MI* maximum intensity, *TTP* time to peak

**Fig. 5:**
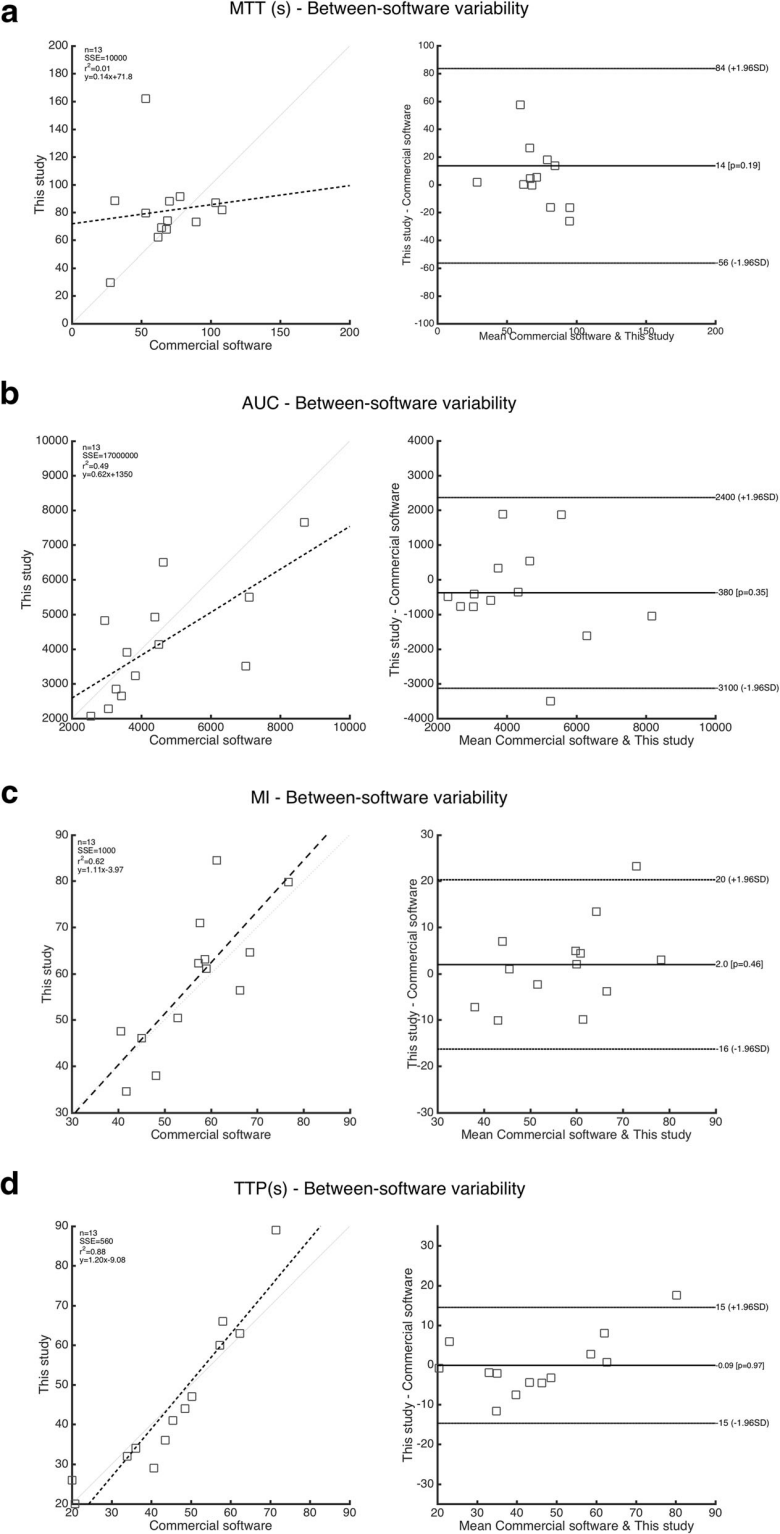
Linear regression and Bland-Altman plots. **a** Mean transit time (s). **b** Area under the curve (a.u.). **c** Maximum intensity (a.u.). **d** Time to peak (s)

The AUC (*r* = 0.70, *p* = 0.007), MI (*r* = 0.79, *p* = 0.001), and TTP (*r* = 0.97, *p* < 0.001) variables presented a significant correlation between the in-house and the commercial tools. Nevertheless, MTT presented significant differences in some patients, with no significant correlation (*r* = 0.21, *p* = 0.48). The linear regression and Bland-Altman graphical comparison of the two methods can be appreciated in Fig. 5. For MTT and AUC, the bias was of 14 s and 380 a.u., respectively, while for MI and TTP were highly close to the line of equality, being 2 a.u. and - 0.09 s. The limits of agreement were also higher for MTT and AUC.

## Discussion

Exciting advances have been produced in the contrast agent field for medical imaging in the past two decades, particularly in the development of compounds with better enhancement, signal properties, pharmacokinetics, and safety. The interaction between these agents and the human body can be actually analysed *in vivo* by US, using new methods that allow the acquisition of images of remarkable quality to monitor organs and lesions in order to detect and characterise disorders with high accuracy. US perfusion techniques are therefore expanding the range of clinical applications of this imaging modality.

Contrast-enhanced US offers important advantages over existing imaging modalities, as it is highly available without any safety issues [24, 25]. However, the clinical value of this perfusion technique is compromised by the relatively large variations in the imaging procedures, protocols, and quantification results due to the lack of standardisation.

In this paper, we have examined gain, dynamic range, and frequency contribution to the variations in contrast behaviour and signal intensity, thus directly influencing the quantitative perfusion measurements. Beyond system settings, which was the goal of the optimisation study, other factors like microbubble-based contrast agent properties and patient management were considered constant [20]. With this optimisation, a standardisation of the acquisition configuration profile that minimised the error in the US perfusion quantitative analysis was achieved.

To our knowledge, a virtual phantom for US perfusion simulation like the described in the present manuscript has not been previously developed. This approach allowed the evaluations of the influence of the system input parameters on the quantification output (*i.e.,* on the TIC) as all other influential factors, like those related to patient conditions and contrast agent properties, were preserved to have the minimum variations between examinations [5, 20]. In the case of patients, the preparation phase was the same across the patients in order to avoid influence of external factors in the results.

The optimum configuration obtained was found to minimise the error of the results while preserving images readability in clinical routine. For all the experiments, the focus was considered to be placed below the region of interest and the mechanical index was fixed to 0.08 to avoid microscopic air bubbles. The mechanical index is correlated to the probability of formation of microscopic air bubbles and is inversely proportional to the square root of the US frequency: by increasing the intensity of the US, mechanical index increases, with a higher probability to have microscopic air bubbles. The focus parameter was specified far from the region of interest, since it significantly modifies the amplitude of the signal, following conventional procedures in US perfusion. Under these optimised conditions, the highest relative error was obtained for MTT, followed by AUC, TTP, and MI, by comparing the values obtained with the previously imposed ones in the virtual phantom creation. Nevertheless, all relative errors were below 1.50%.

After the acquisition standardisation, tests were performed in patients with diagnosed liver hepatocarcinoma. In these *in vivo* studies, it was found that for a proper and correct diagnosis it was necessary to increase the gain to 15 dB for a better detection and visualisation of the lesions and, therefore, a better diagnosis. No other parameters needed to be modified.

The studies were analysed both with the commercial software and with the in-house developed application. Although in the same numerical range, different values were obtained in several patients for the MTT and AUC parameters, while MI and TTP were close to the reference. This variability can be explained by the different strategies that both software tools use for the calculation of the washout, since both MTT and AUC are related to this phase. The Bland-Altman analysis clearly showed that a systematic difference exist between both software tools for MTT and AUC. In the case of our software, MTT was considered as the range of times corresponding to the full width at half maximum of peak enhancement, as usually performed for enhancement analysis in perfusion studies across other modalities like magnetic resonance imaging. For the calculation of the AUC, the whole washout decay curve was considered in our software. End of washout phase was considered when the signal slope was equal to 0 after the arterial peak. The criteria for the algorithm included in the commercial software is unknown, and although it was considered as the reference standard, its use today is only allowed in the research field due to the lack of the 510 (k) clearance from the United States Food and Drug Administration. MI and TTP parameters, which are uniquely calculated from the arterial phase, were highly similar.

Current contrast-enhanced US methods do not consider standardisation of the input parameters. However, they have been shown to induce changes in the calculated perfusion imaging biomarkers. The determination of optimum input parameters allowed robust quantification, providing perfusion parameters useful in a clinical context.

The study presented some limitations that should be commented. Related to the stability of the US contrast agent, preparation, conservation, and administration time affect quantification. Some studies reported a significant incidence of spontaneous gas diffusion phenomena on temporal evolution of the contrast microbubble size [23]. Bubbles have a wide range of distributions in terms of size and coating parameters, which are largely unknown [18, 19]. Second, image preparation and analysis consisted of collecting scan-converted video data and not *Digital Imaging and COmmunications in Medicine* (DICOM) files. Third, due to breathing movement, the liver was slightly displaced during acquisitions, thus introducing some variability in resulting images. Co-registration of motion was applied, but no significant improvements were due to the complexity of the co-registration in the liver. A US spatial registration technique should be proposed as an improvement to our analysis methodology. However, due to the high variability in liver parenchyma, this can be considered a future challenge. As fourth and fifth limitations, the cohort recruited consisted of a small patient sample size and the decision to use a specific commercial software instead of another was based on technology availability at our centre. Finally, the influence of patient size and amount of subcutaneous/visceral fat were not collected in this study. This factor, together with the hepatocarcinoma location within the liver (superficial, closer to the probe or deeper in the liver parenchyma) may have influenced perfusion results due to different expected intensities in the US probe. This topic is now under consideration by our research group.

In conclusion, a US perfusion simulator methodology was engineered by means of creating a digital reference object acting as a virtual phantom. A tool for TIC analysis that was developed in-house permitted the quantification of perfusion properties. Both solutions allowed the evaluation of the optimum US input parameters to minimise variability and obtain a high reproducibility in the perfusion measurements. Our results may add relevant insight into the current knowledge of US perfusion acquisition standardisation and its use in oncology.

## Abbreviations

AUC: Area under the curve
LDRW: Local density random walk
MI: Maximum intensity
MTT: Mean transit time
TIC: Time-intensity curve
TTO: Time to peak
US: Ultrasonography

## References

[1] Parker JM, Weller MW, Feinstein LM et al (2013) Safety of ultrasound contrast agents in patients with known or suspected cardiac shunts. Am J Cardiol 112:1039–1045. 10.1016/j.amjcard.2013.05.042 23816393

[2] Dhamija E, Paul SB (2014) Role of contrast enhanced ultrasound in hepatic imaging. Trop Gastroenterol 35:141–151. 10.7869/tg.201 26012317

[3] Wang XY, Kang LK, Lan CY (2014) Contrast-enhanced ultrasonography in diagnosis of benign and malignant breast lesions. Eur J Gynaecol Oncol 35:415–420. 25118483

[4] Wang S, Yang W, Zhang H, Xu Q, Yan K (2015) The role of contrast-enhanced ultrasound in selection indication and improveing diagnosis for transthoracic biopsy in peripheral pulmonary and mediastinal lesions. Biomed Res Int 2015:231782. 10.1155/2015/231782 PMC4450237 26090391

[5] Green MA, Mathias CJ, Willis LR, et al (2007) Assessment of Cu-ETS as a PET radiopharmaceutical for evaluation of regional renal perfusion. Nucl Med Biol 34:247–255. 10.1016/j.nucmedbio.2007.01.002 17383574

[6] Daghini E, Primak AN, Chade AR, et al (2007) Assessment of renal hemodynamics and function in pigs with 64-section multidetector CT: comparison with electron-beam CT. Radiology 243:405–412. 10.1148/radiol.2432060655 17456868

[7] Martin DR, Sharma P, Salman K, et al (2008) Individual kidney blood flow measured with contrast-enhanced first-pass perfusion MR imaging. Radiology 246:241–248. 10.1148/radiol.2461062129 18096538

[8] Tang MX, Mulvana H, Gauthier T, et al (2011) Quantitative contrast-enhanced ultrasound imaging: a review of sources of variability. Interface Focus 1:520–539. 10.1098/rsfs.2011.0026 PMC3262271 22866229

[9] Gauthier TP, Averkiou MA, Leen EL (2011) Perfusion quantification using dynamic contrast-enhanced ultrasound: the impact of dynamic range and gain on time-intensity curves. Ultrasonics 51:102–106. 10.1016/j.ultras.2010.06.004 20643467

[10] Möller I, Janta I, Backhaus M, et al (2017) The 2017 EULAR standardised procedures for ultrasound imaging in rheumatology. Ann Rheum Dis. 76:1974–1979. 10.1136/annrheumdis-2017-211585 28814430

[11] Pitre-Champagnat S, Coiffier B, Jourdain L, Benatsou B, Leguerney I, Lassau N (2017) Toward a standardization of ultrasound scanners for dynamic contrast-enhanced ultrasonography: methodology and phantoms. Ultrasound Med Biol. 10.1016/j.ultrasmedbio.2017.06.032 10.1016/j.ultrasmedbio.2017.06.032 28779957

[12] Shunichi S, Hiroko I, Fuminori M, Waki H (2009) Definition of contrast enhancement phases of the liver using a perfluoro-based microbubble agent, perflubutane microbubbles. Ultrasound Med Biol 35:1819–1827. doi 10.1016/j.ultrasmedbio.2009.05.013 19713032

[13] Fairbank WM Jr, Scully MO (1977) A new noninvasive technique for cardiac pressure measurement: resonant scattering of ultrasound from bubbles. IEEE Trans Biomed Eng 24:107–110. 10.1109/TBME.1977.326112 892812

[14] Malm S, Frigstad S, Helland F, Oye K, Slordahl S, Skjarpe T (2005) Quantification of resting myocardial blood flow velocity in normal humans using real-time contrast echocardiography. A feasibility study. Cardiovasc Ultrasound 3:16. 10.1186/1476-7120-3-16 PMC1184091 15958173

[15] Arditi M, Frinking PJ, Zhou X, Rognin NG (2006) A new formalism for the quantification of tissue perfusion by the destruction-replenishment method in contrast ultrasound imaging. IEEE Trans Ultrason Ferroelectr Freq Control 53:1118–1129. 10.1109/tuffc.2006.1642510 16846144

[16] Savic RM, Jonker DM, Kerbusch T, Karlsson MO (2007) Implementation of a transit compartment model for describing drug absorption in pharmacokinetic studies. J Pharmacokinet Pharmacodyn 34:711–726. 10.1007/s10928-007-9066-0 17653836

[17] Averkiou M, Lampaskis M, Kyriakopoulou K, et al (2010) Quantification of tumor microvascularity with respiratory gated contrast enhanced ultrasound for monitoring therapy. Ultrasound Med Biol 36:68–77. 10.1016/j.ultrasmedbio.2009.07.005 19900749

[18] Kuenen MP, Mischi M, Wijkstra H (2011) Contrast-ultrasound diffusion imaging for localization of prostate cancer. IEEE Trans Med Imaging 30:1493–1502. 10.1109/TMI.2011.2125981 21402509

[19] Garcia D, Le Tarnec L, Muth S, Montagnon E, Porée J, Cloutier G(2013) Stolt’s f-k migration for plane wave ultrasound imaging. IEEE Trans Ultrason Ferroelectr Freq Control 60:1853–1867. 10.1109/TUFFC.2013.2771 PMC3970982 24626107

[20] Brands J, Vink H, Van Teeffelen JW (2011) Comparison of four mathematical models to analyze indicator-dilution curves in the coronary circulation. Med Biol Eng Comput 49:1471–1479. 10.1007/s11517-011-0845-9 PMC3223587 22095316

[21] Zhou JH, Cao LH, Zheng W, Liu M, Han F, Li AH (2011) Contrast-enhanced gray-scale ultrasound for quantitative evaluation of tumor response to chemotherapy: preliminary results with a mouse hepatoma model. AJR Am J Roentgenol 196:W13-17. 10.2214/AJR.10.4734 21178025

[22] Wei K, Jayaweera AR, Firoozan S, Linka A, Skyba DM, Kaul S (1998) Quantification of myocardial blood flow with ultrasoundinduced destruction of microbubbles administered as a constant venous infusion. Circulation 97:473–483 10.1161/01.cir.97.5.473 9490243

[23] Riascos P, Velasco-Medina J (2005) Efectos Biológicos y Consideraciones de Seguridad en Ultrasonido. Available via https://www.yumpu.com/es/document/view/15350629/efectos-biologicos-y-consideraciones-de-seguridad-en-ultrasonido

[24] de Jong N, ten Cate FJ, Vletter WB, Roelandt JR (1993) Quantification of transpulmonary echocontrast effects. Ultrasound Med Biol 19:279–288 10.1016/0301-5629(93)90100-3 8346602

[25] Quaia E (2011) Assessment of tissue perfusion by contrast-enhanced ultrasound. Eur Radiol 21:604–615. 10.1007/s00330-010-1965-6 20927527

